# Continuous Remimazolam Administration by Gastroenterologists for Endoscopic Sedation

**DOI:** 10.3390/medicina62040723

**Published:** 2026-04-10

**Authors:** Tanya M. Bisseling, Angela van Zuuk, Michiel Vaneker, Hennie Hukker, Cariline Roosen, Jasmijn Olde, Marjolijn Duijvestein, Geert J. Bulte, Lucas T. van Eijk, Jörgen Bruhn

**Affiliations:** 1 Department of Gastroenterology and Hepatology, Radboud University Medical Center, 6525 GA Nijmegen, The Netherlands; 2 Department of Anesthesiology, Pain and Palliative Medicine, Radboud University Medical Center, 6525 GA Nijmegen, The Netherlands

**Keywords:** remimazolam, continuous administration, endoscopy, gastroenterology

## Abstract

*Background and Objectives:* Gastrointestinal (GI) endoscopy requires safe and effective sedation. Remimazolam, an ultra-short-acting benzodiazepine, may offer advantages over traditional sedatives like midazolam and propofol, including rapid onset, short half-life, and a favorable safety profile. This study evaluates the feasibility, safety, and patient satisfaction of continuous remimazolam infusion administered by trained gastroenterologists for GI endoscopy. *Materials and Methods:* This prospective registry included patients with ASA physical status I and II undergoing standard endoscopic procedures. Continuous remimazolam sedation was administered, with boluses given as needed. Vital signs were monitored, and patient satisfaction was assessed before and after the procedure using standardized questionnaires. *Results:* A total of 159 procedures were performed in 141 patients. Sedation was successful in all patients, with a mean induction dose of 7.1 mg and total infusion of 15.1 mg. Recovery time averaged 3.3 min. Adverse events, including transient hypotension and hypoxia, occurred in 11.3% of patients but were easily managed. Most patients (97%) reported sufficient comfort, with an average satisfaction score of 8.1/10. *Conclusions:* Continuous remimazolam infusion administered by trained gastroenterologists is a safe and effective alternative to traditional propofol sedation for GI endoscopy. It offers stable sedation, rapid recovery and high patient satisfaction, potentially reducing anesthesiology workload and improving procedural efficiency. Further studies are needed to confirm these findings in broader patient populations.

## 1. Introduction

Gastrointestinal (GI) endoscopy comprises invasive diagnostic and therapeutic procedures. Colonoscopies are among the most frequently performed procedures, with approximately 110,000 procedures performed annually in the Netherlands and 15 million in the USA. For patients with an increased risk of GI cancers—such as those with hereditary cancer syndromes or chronic inflammatory bowel disease (IBD)—regular endoscopic surveillance is essential. The routine method for sedation by the gastroenterologist during endoscopy involves intravenous bolus administration of a combination of a benzodiazepine, such as midazolam, with an opioid, such as fentanyl, inducing moderate sedation. Still, a considerable proportion of patients experience discomfort. Such patients may then be referred for endoscopy under procedural sedation and analgesia (PSA), which encompasses titration to a deeper level of sedation, commonly using propofol. Use of PSA has increased over the past years and continues to do so. Although this provides effective and rapid sedation and analgesia, it relies on highly trained anesthesia care professionals in most countries, due to the small therapeutic range of propofol and the lack of an antidote. Moreover, patients usually need to be pre-screened in the anesthesia outpatient clinic. These requirements prolong waiting times, increase healthcare expenditure and environmental burden, and may delay surveillance intervals, increasing the risk of interval cancers in high-risk patients. Given the increasing demand for PSA, there is a clear need for suitable and safe alternatives.

Since 2021, the ultra-short-acting benzodiazepine remimazolam has become available for procedural sedation in Europe. In 2023 it was also registered for the induction and maintenance of general anesthesia. Compared with midazolam, remimazolam exhibits approximately threefold faster clearance and sevenfold shorter systemic retention [1]. The drug is associated with a lower incidence of hypotension and respiratory depression compared with propofol [2,3,4,5,6,7,8] and is not painful upon injection. Moreover, remimazolam’s short half-life allows for more precise titration compared with midazolam, and its effect can be quickly and fully reversed with flumazenil. According to the label, for sedation during endoscopic procedures, remimazolam is administered as an intravenous bolus. If necessary, additional boluses can be administered. However, its pharmacokinetics have also been well-documented for continuous infusion use [9]. As such, a more stable and longer-lasting sedation can be achieved, which allows its application for longer endoscopic procedures. This was observed in a randomized study of patients undergoing endoscopic mucosal resection in the esophagus [5]. It has also been safely used during other procedures in adolescents [6,7] and elderly people [8].

Studies have shown that patients recover more quickly from remimazolam sedation compared with propofol sedation [9,10]. Additionally, multiple case reports describe its successful and safe use in complex patients for whom traditional anesthesia or sedation posed a high risk, including patients with severe aortic valve stenosis, myotonic dystrophy, ALS, and Takayasu’s arteritis, and who are post-liver transplantation [11,12,13,14,15].

Use of remimazolam as an alternative sedative may reduce healthcare and patient burden, costs [16], and the environmental impact of endoscopic sedation. It may offer a safe, comfortable and easily titratable method of sedation during endoscopic procedures, potentially reducing the need to refer patients to a PSA program. Moreover, its use in selected patients is expected to result in high patient satisfaction. This prospective registry was conducted as a proof of concept to evaluate the feasibility, safety and patient satisfaction associated with continuous remimazolam sedation by the gastroenterologist during gastrointestinal endoscopy.

## 2. Materials and Methods

### 2.1. Study Design

The study was designed as a prospective registry of data gathered after implementation of remimazolam as standard of care in the endoscopy unit of the Radboud University Medical Center, Nijmegen, the Netherlands. This explicitly was not a comparative study in which remimazolam was compared to other sedatives, but rather a prospective evaluation of the feasibility, safety and practicality of continuous remimazolam administration by trained gastroenterologists in an endoscopic setting. Prior to incorporation in standard endoscopy practice, endoscopy personnel had received additional hands-on training by an anesthesiologist, and a pilot phase under the direct supervision of an anesthesiologist had been completed. Investigations were conducted in accordance with the principles outlined in the Declaration of Helsinki (1975, revised in 2013). This project was evaluated by the responsible medical ethics committee (CMO Arnhem-Nijmegen, file number 2024-17707). No formal judgment was deemed necessary due to the observational nature of the study. However, in conjunction with the European General Data Protection Regulation, all participating patients were asked for verbal informed consent for the use of their data.

### 2.2. Patients and Procedures

Patients with an ASA physical status primarily classified as I–II were eligible for inclusion. ASA classification was assigned during routine pre-procedural clinical assessment. As this study reflects daily clinical practice, no post hoc exclusions were performed when deviations from the intended ASA classification were identified retrospectively. Exclusion criteria were BMI ≥ 35, Mallampati score ≥ 3, and more complex endoscopic procedures such as piecemeal EMR, ESD, ERCP, or dilations. Pre-procedural screening of all patients was performed by specialized nurses from the gastroenterology department, equal to patients receiving midazolam sedation. It is important to note that, while gastroenterologists were responsible for the sedation, the screening was conducted by specialized nurses from the gastroenterology department rather than anesthesiologists, addressing the concern about the roles of different medical professionals in the sedation process. Most procedures were colonoscopies, mostly for surveillance of inflammatory bowel disease (IBD), (hereditary) familial colon cancer or adenomas. Remimazolam treatment was offered to those fulfilling the inclusion criteria, even if they had received propofol sedation during a previous endoscopy. All procedures were performed by experienced gastroenterologists. Routine interventions such as forceps biopsy, cold-snare or hot-snare polypectomy, or APC were performed when necessary.

Sedation was performed using continuous i.v. infusion of remimazolam solution with a concentration of 1 mg ml^−1^, starting at a dose of (actual body weight × 0.5) mg h^−1^, combined with an induction bolus administration of (weight × 0.075–0.10) mg, rounded to whole milligrams (e.g., a patient of 80 kg received a dose of 6 mg). Additional boluses of 1–4 mg were administered when the level of sedation was inadequate. The rate of continuous administration was adjusted stepwise to a maximum of 1 mg kg^−1^ h^−1^ as needed. Alfentanil was used at induction in a dose of 0.25–0.5 mg, and additional boluses of 0.25 mg could be administered during the procedure at the discretion of the gastroenterologist. Sedation and monitoring during the procedures were managed by specialized gastroenterology nurses trained in sedation, under the direct supervision of the gastroenterologists.

Oxygen was supplemented through a nasal canula at a flow rate of 2 L min^−1^ as a standard precaution. Patients were continuously monitored by ECG and pulse oximetry, and intermittent measurements of non-invasive blood pressure.

After the procedure and after emergence from sedation, patients were transported to the recovery room of the endoscopy unit and supervised by dedicated nurses. Discharge of patients to their home environment was based upon the Aldrete recovery score of ≥9. Relevant time intervals were measured, and patient satisfaction was reported by endoscopy nurses according to the Gloucester Comfort Score (GCS) [17] and questionnaires both before and after the procedure.

### 2.3. Data Collection and Statistical Analysis

Collected patient-level variables comprised demographics, relevant medical history, and use of medications that may potentiate sedative or respiratory effects, such as opioids, antipsychotics, sedating antidepressants, and benzodiazepines. Chronic benzodiazepine use was specifically recorded due to potential pharmacological tolerance. Previous endoscopy experience of the patients was also recorded. Procedure-related data included endoscopy type and duration, interventions, bowel preparation quality (Boston Bowel Preparation Scale), patient comfort (GCS), vital signs (systolic/diastolic blood pressure, heart rate, oxygen saturation, and MAP), and all administered medications with timing and dose adjustments. A saturation < 90% as well as a MAP < 60 mmHg were considered abnormal. A descriptive stratified analysis was performed comparing patients who received supplemental alfentanil with those who did not. Adverse events were categorized according to their clinical severity, based on the need for intervention and patient impact: mild: event resolved spontaneously without intervention; moderate: required simple interventions (e.g., IV fluids for hypotension, supplemental oxygen for desaturation); or severe: required invasive intervention, vasopressors, or ICU admission. Analyses of patient-reported outcomes were performed on completed surveys only (complete-case analysis). Missing survey data were not imputed.

Statistical analysis was performed using IBM SPSS Statistics version 29. Descriptive statistics summarized patient characteristics, procedural data, and safety outcomes. Continuous variables were reported as means with standard deviations, and categorical variables as frequencies and percentages. Pre- and post-procedural differences in comfort, anxiety, and pain were analyzed using the Wilcoxon signed-rank test. A *p*-value < 0.05 was considered statistically significant.

To explore predictors of recovery time, multiple linear regression was conducted with age and body weight as independent variables. Model performance was assessed using R^2^, adjusted R^2^, and ANOVA. Vital sign visualizations were created using GraphPad Prism version 9.1.1.

## 3. Results

### 3.1. Patient Characteristics

Patient characteristics are described in Table 1. A total of 159 endoscopies were performed in 141 unique patients under remimazolam sedation. In 18 patients, simple upper endoscopy was combined with sigmoidoscopy, pouchscopy or colonoscopy during the same session. The majority of patients were classified as ASA I–II. Four patients were classified as ASA III at the time of the procedure. The mean age of the cohort was 48.5 years (SD ± 16.6), and the majority was female (n = 94, 66.7%).

Nine patients (6%) had no prior history of endoscopic procedures. One hundred and thirty-two patients (94%) previously underwent one or more endoscopic procedures under propofol sedation, after midazolam sedation had proven to be inadequate or unsuccessful. Most participants (n = 105, 74.5%) were referred for surveillance in the context of familial tumor syndromes. Procedural data are described in Table 2. Mean procedure duration was 24.9 ± 11.6 min. All planned endoscopies were completed successfully.

### 3.2. Sedation

**Figure 1:**
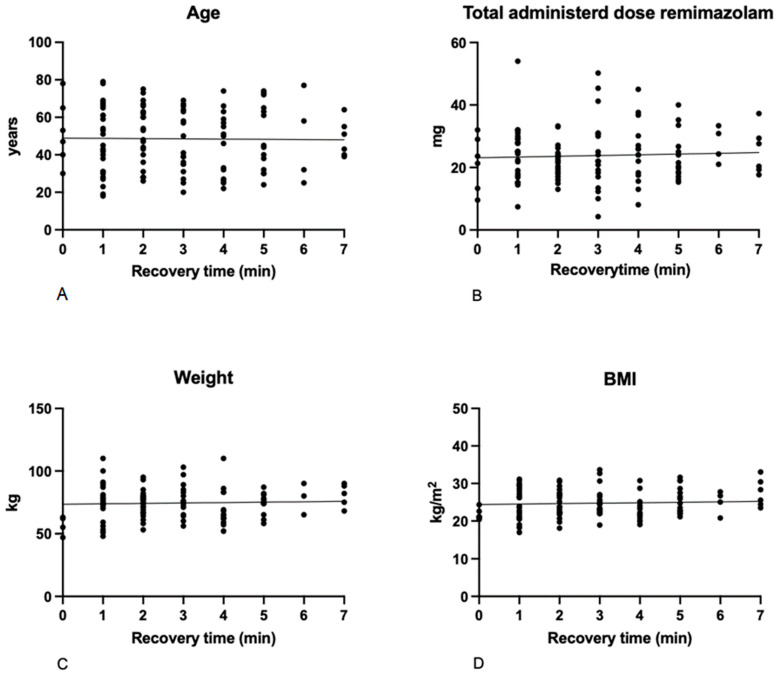
Scatterplots of the relationships between patient characteristics and recovery time. (**A**): Relationships between awakening time and age (R^2^ = 0.00, F = 0.16, *p* = 0.69); (**B**): total administered dose of remimazolam (R^2^ = 0.02, F = 2.24, *p* = 0.14); (**C**): weight (R^2^ = 0.01, F = 1.14, *p* = 0.29); and (**D**): body mass index (BMI,: R^2^ = 0.02, F = 2.70, *p* = 0.1).

Sedation was initiated with an average remimazolam dose of 7.1 ± 1.9 mg, supplemented by 0.4 ± 0.1 mg alfentanil. Sedation was successful in all patients. During the procedure, a total of 15.1 ± 6.9 mg remimazolam was administered per continuous infusion. In 11 patients, the infusion rate was increased during the procedure, while in 8 it was decreased. Additional remimazolam boluses of 1–4 mg were administered in 90 patients (63.8%). Endoscopy could be started 3 ± 2 min after starting remimazolam administration. After stopping the remimazolam infusion, it took 3.3 ± 2.4 min for patients to wake up. Awakening time was not associated with age, weight/BMI or administered total dose of remimazolam (Figure 1).

### 3.3. Safety and Adverse Events

**Figure 2:**
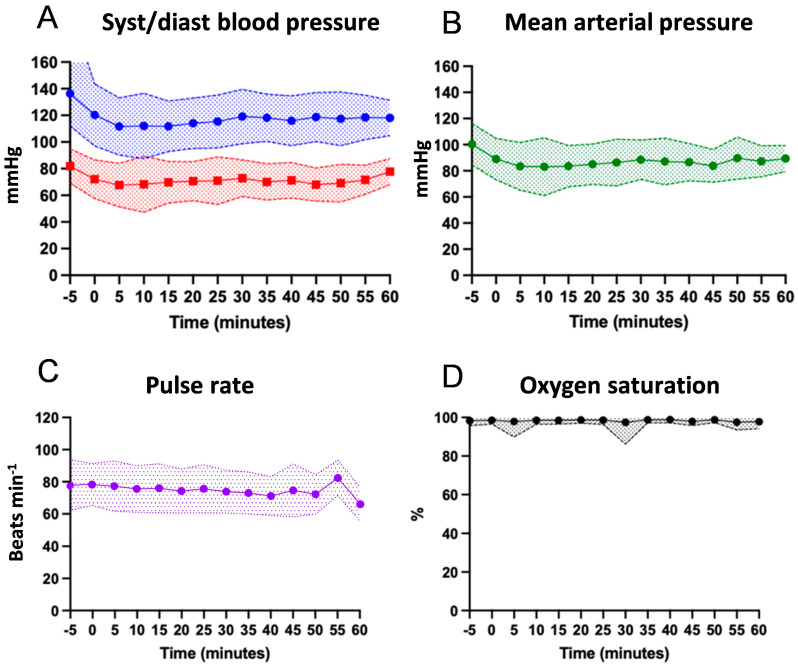
Vital signs during endoscopy. (**A**): Systolic and diastolic blood pressure; (**B**): Mean arterial pressure; (**C**): heart rate; (**D**): oxygen saturation. All data are expressed as mean ± SD.

Vital signs, including systolic and diastolic blood pressure, MAP, heart rate, and oxygen saturation, generally remained stable throughout the procedures (Figure 2A–D).

A total of 16 patients (11.3%) experienced sedation-related adverse events, forming the adverse-event subgroup. All underwent colonoscopy, except for two who had combined duodenoscopy and sigmoidoscopy. None used medications known to interact with remimazolam. A mean arterial pressure (MAP) < 60 mmHg was observed in 16 patients. Hypotensive episodes occurred predominantly in patients receiving additional alfentanil (15/16 events), whereas only one episode occurred in patients who did not receive alfentanil. While exact minute-by-minute timing was not captured, these 15 hypotensive episodes occurred shortly after alfentanil administration, consistent with a temporal association.

In these 16 patients, the MAP decreased to 54.0 ± 4.7 mmHg over a median duration of 5 min. In four patients (2.8%), it lasted 10–20 min and required intervention, classifying these as moderate adverse events. Apart from lowering the remimazolam perfusion dose, three patients received a fluid bolus of 250 mL 0.9% NaCl intravenously, and one was managed by adjusting bed positioning. All patients recovered uneventfully. Transient hypoxia (oxygen saturation <90%) occurred in two patients (1.4%), each at a single time point (lowest value: 85%), which could be easily managed by increasing the oxygen flow to 4 L/min, also classifying these adverse events as moderate. No patients required airway maneuvers like jaw thrust or insertion of an oral airway device. No allergic reactions, bradycardia, or apnea were reported.

### 3.4. Patient Experience

In total, 126/141 (89%) completed a survey before and after the endoscopy. Of the 115 patients who underwent a colonoscopy, in 92 (92.0%), no to mild discomfort was reported by endoscopy nurses according to the GCS. In four patients, significant discomfort, and in four patients, extreme discomfort was reported. Still, 97 (97%) experienced sufficient comfort during remimazolam sedation, whereas four preferred to go back to propofol sedation the next time.

Where 36/126 reported nervousness and anxiety beforehand due to the new sedation method, afterwards, the average patient-reported comfort during endoscopy was 8.1 ± 1.6 on a scale of 1 to 10.

## 4. Discussion

This study investigates the feasibility, safety, and efficacy of continuous remimazolam infusion administered by trained gastroenterologists without anesthesiology support for procedural sedation during gastrointestinal endoscopy. Our findings demonstrate that remimazolam sedation can be safely and effectively maintained per continuous infusion, with high patient satisfaction and no major safety concerns. This approach offers a promising alternative to traditional sedation protocols, likely increasing the success rate compared to midazolam sedation, while on the other hand reducing the need for anesthesiologist-led propofol sedation, decreasing healthcare costs, and improving patient throughput in endoscopy units.

One of the main advantages of remimazolam is its rapid onset and short context-sensitive half-life [18], making it particularly well-suited for titratable sedation during endoscopic procedures. In our cohort, sedation was consistently achieved quickly, and recovery times were rapid, supporting more efficient patient flow. These pharmacokinetic properties could offer logistical and economic benefits, particularly in high-volume endoscopy settings where quick turnover is essential. Continuous infusion of remimazolam allows for more stable sedation, reducing fluctuations in sedation depth and enabling careful titration to the desired sedation level. This method requires minimal intervention once the target depth is reached, which contrasts with the repeated bolus doses of remimazolam typically required for longer procedures.

In terms of safety, remimazolam demonstrated a favorable profile, with low rates of respiratory and cardiovascular depression. Although propofol remains the gold standard for deep sedation, particularly in anesthesiologist-led settings, remimazolam offers a safer alternative for moderate-to-deep sedation administered by non-anesthesiologists without compromising procedural efficacy. Importantly, no major adverse events requiring anesthesiologist intervention were observed, and the incidence of hypoxia and hypotension was low. This supports the growing body of evidence suggesting that remimazolam may be a safer alternative to traditional sedatives like midazolam or propofol, particularly for patients with comorbidities.

The analysis of the episodes of hypotension observed in our study reveals a temporal association with the administration of alfentanil. The temporal association suggests that opioid co-administration may have contributed to hemodynamic events rather than remimazolam itself. This observation is consistent with the known synergistic cardiovascular effects of benzodiazepines and opioids. Importantly, sedation with remimazolam alone appeared hemodynamically stable in most patients. As opioid supplementation was clinically indicated and not protocol-driven, causal inference cannot be established. The potential contribution of opioid co-administration, rather than remimazolam alone, should be carefully considered when interpreting the safety findings.

Given the known hemodynamic effects of opioids, future studies should consider isolating the effects of remimazolam by using alternative analgesic agents or by assessing the contribution of opioids more specifically. This would help in better understanding the safety profile of remimazolam in combination with different analgesics.

The pharmacokinetic advantage of remimazolam over midazolam is particularly notable, as it is metabolized independently of organ function [19], potentially reducing variability in sedation quality and recovery time. This makes it especially useful for elderly patients or those with underlying health conditions. Furthermore, continuous infusion provides a more stable sedation plane compared to bolus protocols, minimizing patient responsiveness and the need for rescue medications or procedural interruptions. Our findings are in line with a recent study of Tian et al. [20] investigating continuous remimazolam administration in combination with alfentanil to elderly patients undergoing colonoscopic polypectomy. In their trial, a remimazolam maintenance dose of 0.5 or 0.75 mg kg^−1^ h^−1^ resulted in less hypotension and less respiratory depression compared with propofol. Remarkably, significant increases in awakening time and PACU length of stay were observed, which is in contrast to our findings and warrants further investigation. Possibly, elderly patients may be more at risk for prolonged sedative effects, although remimazolam’s pharmacokinetic properties were previously reported not to be different in elderly people [21]. The results of Tian et al. and ours indicate that remimazolam, when titrated and administered continuously, provides a reasonable compromise between sedation depth and patient safety, supporting its use for both moderate and deep sedation. To the best of our knowledge, no other trials using continuous administration of remimazolam for procedural sedation have been published, despite its great potential.

A key feature of this study is that sedation was administered exclusively by trained gastroenterology teams, reflecting a potential shift in procedural sedation practices. This aligns with current trends towards non-anesthesiologist-administered sedation protocols [22], provided that proper training, continuous monitoring, and institutional frameworks are in place.

Several limitations must be acknowledged. The study was conducted at a single center with experienced endoscopists, which may limit the generalizability of the findings. Although inclusion primarily targeted ASA I–II patients, a small number of ASA III patients were included. As this reflects routine clinical practice and the proportion was limited, this is unlikely to have significantly influenced the overall findings. Excluding these patients retrospectively could have introduced selection bias in this observational cohort. Additionally, the patient population was predominantly female, and those with higher ASA classifications were excluded from the study, potentially biasing the results. The non-comparative nature of the study and the absence of a control group, such as a propofol- or midazolam-based sedation cohort, should also be explicitly noted as limitations. However, direct comparison of remimazolam with a control group was not the intention of the study. We show that continuous remimazolam sedation by trained gastroenterologists is safe and effective, and as such offers an alternative to referral for propofol sedation, rather than showing superiority or inferiority.

Other limitations are that patient-reported outcomes may reflect post-procedural relief rather than intra-procedural comfort, and the five-minute intervals at which vital signs were recorded may have missed transient fluctuations. The precise timing of hypotensive episodes relative to alfentanil administration was not systematically recorded. Nevertheless, the temporal relationship observed supports the descriptive finding that most hypotensive events occurred following alfentanil administration rather than remimazolam alone.

Furthermore, objective and standardized sedation depth measurements, such as the Modified Observer’s Assessment of Alertness/Sedation (MOAA/S) or bispectral index monitoring, are lacking. Incorporating such measures in future studies would help enhance the comparability of the results. Furthermore, monitoring sedation depth may aid in safety, as moderate sedation carries the potential for unintended progression to deeper sedation, which has important implications for patient safety, monitoring, and staffing. In that respect, remimazolam has the advantage of a better safety profile, showing lower incidence of hypoxia and hypotension at the same sedation level compared with propofol [23]. In addition, the gastroenterologists involved in this study were specifically trained to manage sedation with continuous remimazolam, and sedation was carefully titrated according to patient response. Furthermore, sedation was administered with close monitoring, including ECG, pulse oximetry, and non-invasive blood pressure measurement, which ensured the safety of our patients throughout the procedures.

As a final limitation, the absence of time-stamped procedural events (e.g., endoscope looping or colonic insufflation) complicates the interpretation of the findings.

Although rare, adverse reactions to remimazolam have been described, including the risk of delayed awakening, re-sedation after initial recovery and anaphylaxis [24]. None of our patients had any of these reactions. Still, the risk of such events may be higher compared with midazolam or propofol, which may be relevant when using remimazolam in routine care.

The cost-effectiveness of this approach is another potential benefit, as it may reduce the need for anesthesiologic care and shorten waiting lists for patients who require procedural sedation. Given that remimazolam infusion can be administered by trained endoscopy staff, it reduces the burden on anesthesia resources and improves efficiency. However, it is important to emphasize that patient safety remains the primary consideration. While propofol is a commonly used sedative in endoscopy, it is associated with risks such as hypotension, respiratory depression, and apnea, especially in settings without anesthesiology support. These risks are particularly concerning in outpatient settings where anesthesiologists may not be available for continuous monitoring and intervention. Remimazolam offers a more favorable safety profile, with rapid onset and offset of action and a lower incidence of respiratory depression and hypotension compared to propofol. This makes it a promising alternative, particularly in situations where anesthesiology support is not available or not required. While one of the benefits of remimazolam is that it can be managed by trained gastroenterology nurses under the supervision of gastroenterologists, the primary goal of this study was to evaluate the safety and feasibility of the continuous administration of remimazolam, rather than simply to reduce costs or eliminate the need for anesthesiologists.

Future studies should further evaluate the safety, efficacy, and economic impact of remimazolam, especially in high-volume centers. It will also be important to assess outcomes in patients with obesity, multiple comorbidities, or polypharmacy, and to compare remimazolam directly with propofol in matched cohorts.

## 5. Conclusions

Continuous remimazolam infusion administered by trained gastroenterologists without anesthesiology support appears to be a safe and effective alternative to traditional sedation practices for gastrointestinal endoscopy. Its precise titration and rapid recovery suggest it may be a promising option for moderate-to-deep sedation, with notable advantages in terms of cost, throughput, and environmental impact. With appropriate training and monitoring protocols, this approach could expand access to safe and effective procedural sedation in endoscopy units worldwide. Further research, particularly multi-center randomized controlled trials, is needed to confirm these findings and fully explore the potential of remimazolam in diverse patient populations and clinical settings.

## Figures and Tables

**Table 1:** Patient characteristics.

Variables		Mean ± SD or n (%)
Age (yr)		48.5 ± 16.6
Gender	Male	47 (33.3)
	Female	94 (66.7)
BMI (kg/m^2^)		25.1 (3.9)
ASA classification	I	51 (36.9)
	II	86 (61.0)
	III	4 (2.8)
Comorbidities	None	57 (40.4)
	Hypertension	16 (11.3)
	Diabetes mellitus	9 (6.4)
	Cardiovascular disease	11 (7.8)
	COPD	1 (0.7)
	OSAS	3 (2.1)
	Chronic kidney disease	1 (0.7)
	Liver disease	2 (1.4)
	Neurological disorders *	14 (9.9)
	Other	70 (49.6)
Previous abdominal surgery		52 (36.9)
Use of medication affecting sedation	None	127 (90.0)
	Benzodiazepines	5 (3.5)
	Opioids	5 (3.5)
	Antihistaminics	4 (2.8)
Reason for endoscopy	Hereditary cancer	105 (74.5)
	IBD	27 (19.1)
	Other	9 (6.4)

COPD: Chronic obstructive pulmonary disease; OSAS: obstructive sleep apnea syndrome; IBD: inflammatory bowel disease. * Peripheral neuropathy, carpal tunnel syndrome, pyramidal tract syndrome.

**Table 2:** Procedural data.

Variables (n = 141)		Mean ± SD or n (%)
Type of endoscopy	Duodenoscopy	32 (22.7)
	Colonoscopy	115 (81.6)
	Sigmoidoscopy	9 (6.4)
	Pouchscopy	2 (1.4)
	ERCP	0 (0.0)
	Endoscopic ultrasound (EUS)	1 (0.7)
Intervention during endoscopy (78 interventions in 73 pt)	None	68 (48.2)
	Biopsy	44 (31.2)
	Cold-snare polypectomy	28 (19.9)
	Electrocoagulation polypectomy	2 (1.4)
	Dilation	1 (0.7)
	FNA/FNB	1 (0.7)
	Other	2 (1.4)
Administered medication	Induction dose of alfentanil (mg)	0.4 ± 0.1
	Patients receiving additional alfentanil during procedure (n)	51 (36.2)
	Induction dose of remimazolam (mg)	7.1 ± 1.9
	Patients receiving additional remimazolam bolus 1–4 mg (n)	90 (63.8)
	Cumulative continuous remimazolam dose (mg)	15.1 ± 6.9
Adaptation infusion rate	Up	11 (7.8)
	Down	8 (5.7)
Waiting time to start endoscopy after first administration (min)		3.0 ± 2.0
Time to awakening after stopping remimazolam (min)		3.3 ± 2.4
Sedation-related side effects	MAP < 60 mmHg	16 (11.3)
	Fluid challenge required	3/16
	oxygen saturation < 90%	2 (1.4)
	Allergy/anaphylactic reaction	0 (0.0)
**Colonoscopy specific (n = 115)**		
BBPS	≤6	4 (3.5)
	≥7	111 (96.5)
GCS	1	36 (31.3)
	2	46 (40.0)
	3	22 (19.1)
	4	4 (3.5)
	5	4 (3.5)
	Unknown	3

BBPS: Boston bowel preparation score; GCS: Gloucester comfort scale. % are expressed as a percentage of 141 patients.

## Data Availability

Data are available upon reasonable request.

## Abbreviations

The following abbreviations are used in this manuscript:

GI	Gastrointestinal
IBD	Inflammatory bowel disease
PSA	Procedural sedation and analgesia
ASA	American Society of Anesthesiologists
GCS	Gloucester Comfort Score
MAP	Mean arterial pressure
